# Antidepressants synergize with chemotherapy against cancer stem cells

**DOI:** 10.18632/aging.100848

**Published:** 2015-11-18

**Authors:** Gerry Melino

**Affiliations:** Department of Experimental Medicine and Surgery, University of Rome "Tor Vergata", 00133 Rome, Italy

**Keywords:** ITCH, p73, p63, p53, cancer, stem cells

In a recent report [1], the antidepressant drug desmethylclomipramine has shown an interesting synergistic effect with cisplatin, gemcitabine or placlitax on lung cancer stem cells. The fact that antidepressant drugs potentiate chemotherapy in poorly responding cancer is of major relevance, and opens up potential innovative therapeutic effects. But what are the underlying molecular mechanisms for this therapy? Can this be transferred to other poorly responding cancers?

We originally identified the HECT containing ITCH as the ubiquitin E3 ligase responsible for the degradation of p63 and p73, and, based on that, we performed an high throughput (HTS) screening using an ELISA-based HTS with purified recombinant proteins and GST-auto-ubiquitylation as readout for ITCH activity [2]. The automated HTS on a 22,000 compound library was robust, according to an average recorded Z’ of 0.7 (range 0.5–0.7), and resulted in the identification of 46 compounds showing < 50% residual activity, 20 of which were confirmed at single point, and 6 of these showed a dose-dependent inhibition of ITCH. The most active validated inhibitors were the antidepressant drugs clomipramine, norclomipramine and their active metabolite desmethylclomipramine [2]. Interestingly, the validation and analysis of the results revealed clomipramine as a regulator of autophagy [2–3]. This was an intriguing observation as clomipramine has been used for over 40 years for the treatment of patients with psychiatric disorders, with a long-standing record of tolerance and safety. Antidepressants are known to act on the serotonin uptake, so the inhibition of an E3 ligase would suggest that either there is an alternative mode of action, or that an ITCH-related E3 ligase is involved in the process, for example as a regulator of the receptor or of the intracellular recycling pathway. Still, the regulation of autophagy opens interesting opportunities for cancer therapy based on the evidence that autophagy inhibitors potentiate the effect of chemotherapy. Indeed several clinical trials are under way in phase I-II using chloroquine to potentiate chemotherapy (e.g. http://ClinicalTrials.gov Identifier: NCT01510119, NCT01480154, NCT01023477, NCT02421575, NCT01649947). *In vitro*, clomipramine does potentiate the effect of chemotherapeutic agents on different bladder, breast, prostate cancer cell lines [2]. However, to be more effective, this effect should be tested on cancer stem cells. And indeed, the work of Bongiorno-Borbone et al. [1] revealed that clomipramide compounds have a significant effect on the growth properties of lung cancer stem cells isolated from non-small-cell lung cancers’ surgical specimens. Desmethylclomipramine, by interfering with the autophagic flux and blocking the degradation of the autophagic cargo, decreases the stemness potential of cancer stem cells and increases the cytotoxic effect of conventional chemotherapeutic agents on lung cancer [1]. To check whether this effect is due to a selective inhibition of ITCH itself and not to another off target effect, including the inhibition of other HECT-containing E3 ubiquitin ligases, the authors have silenced ITCH. The results indicate that the silencing of ITCH is sufficient to phenocopy the effect of clomipramide. Moreover, the analysis of the expression of ITCH in two distinct data sets of lung adenocarcinoma (GSE31210 and GSE11969) demonstrates that the expression of ITCH is a significant negative prognostic marker, affecting long term Kaplan-Maier survival.

Recent data indicate a complex interplay among p73, p53, mdm2 and HIF-1α in lung cancer [4–5] affecting cancer cell metabolism and cell fate [6–7]. Clearly the consequences of the p53 family inactivation are not limited to the initial loss of genome integrity, i.e. tumour initiation, but also to the tumour micro-environment and tumour progression, where hypoxia and hypoxia-inducible factors (HIFs) are major players in the physiological response to low oxygen. The hypoxic microenvironment causes the cancer cells to co-opt HIF-dependent processes, providing all of the required features for cancer progression. The HIFs, in conjunction to the p53 family, coordinate the transcriptional program required to acquire pro-angiogenic, invasive, and metastatic properties, as well as metabolic adaptations and stemness, which collectively constitute the lethal cancer phenotype. By regulating ITCH and inhibiting autophagy, antidepressant drugs may offer a novel way for safe and effective therapies.

**Figure 1:**
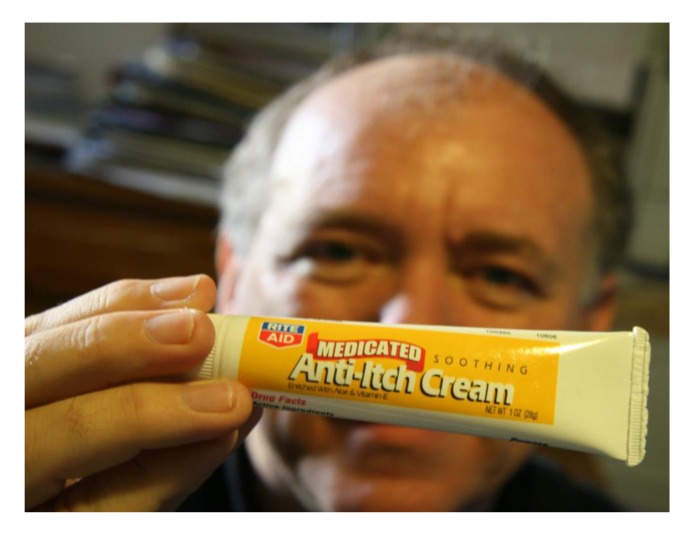
Antidepressants synergize with chemotherapy against cancer stem cells.
